# Hearing Status and Ventilation Tube at Time of Palatoplasty in Cleft Lip and Palate Patients: A Retrospective Study

**DOI:** 10.3390/medicina59030513

**Published:** 2023-03-06

**Authors:** Antonio Frisina, Katherine Piacentile, Andrea Frosolini, Roberto Saetti, Ugo Baciliero, Andrea Lovato

**Affiliations:** 1 Otorhinolaryngology Unit, Department of Surgical Specialties, Valdagno Civil Hospital, 36078 Valdagno, Italy; 2 Maxillofacial Surgery Unit, Department of Surgical Specialties, Vicenza Civil Hospital, 36100 Vicenza, Italy; 3 Maxillofacial Surgery Unit, Department of Medical Biotechnologies, University of Siena, 53100 Siena, Italy; 4 Otorhinolaryngology Unit, Department of Surgical Specialties, Vicenza Civil Hospital, 36100 Vicenza, Italy

**Keywords:** otitis media with effusion, cleft lip and palate, ventilation tube, hearing loss, outcome

## Abstract

*Background and Objectives*: There is no consensus regarding the indications for and timing of ventilation tube (VT) insertion in cleft lip and palate (CLP) patients. Our aim was to search for clinical and surgical (i.e., VT insertion) characteristics that influence the hearing status in CLP. *Materials and Methods*: We reviewed the hearing outcome of consecutive CLP cases operated on at a single referral center. Univariate and multivariate analysis were applied as appropriate. *Results*: We included 285 consecutive CLP patients, 109 female and 176 male; the mean age at last follow-up was 16.2 years. Unilateral CLP was found in 249 cases and bilateral CLP in 36. Early VTs (i.e., at the time of hard palate surgery) were applied in 75 (26.3%) patients. Late VTs (i.e., after hard palate surgery during follow-up) were applied in 69 (24.2%) children, at a mean age of 6.7 years old. Hearing loss (pure-tone average > 20 dB) was found in 114 (40%) CLP patients at last available follow-up (mild hearing loss in 96 patients, moderate in 18). In univariate and multivariate analyses, we found that only the absence of early VT insertion (*p* = 0.0003; OR = 18.2) was an independent prognostic factor of hearing loss in CLP patients. Furthermore, when early VTs were not inserted, there was a high risk of late VT (*p* = 0.002; OR 13.6). *Conclusions*: According to our results, the absence of VT insertion at the time of hard palate surgery is an independent prognostic risk factor of hearing loss in CLP patients. Early VT placement in CLP patients may prevent hearing loss and related consequences. These findings should be tested in a large, randomized clinical trial.

## 1. Introduction

Cleft lip and palate (CLP) in children is frequently complicated by otitis media with effusion (OME), an accumulation of fluid within the middle ear, due to Eustachian tube dysfunction, with an incidence ranging from 74.3% to around 90% in various reports [1,2]. The sequelae of OME also include perforation, adhesion, and retraction of the tympanic membrane, as well as chronic changes such as myringosclerosis or tympanosclerosis [3]. These changes can lead to mild or moderate hearing impairment that may be permanent later in life, especially for the high frequencies that are critical for speech recognition [4]. The best management for OME has still to be defined. Recent international guidelines indicated CLP as a high risk factor for developing OME and strongly recommended against the use of steroids, antibiotics, and antihistamine drugs, but suggested clinical surveillance and ventilation tube (VT) insertion in selected cases [5,6]. Treating OME with VT is one of the most common procedures to prevent the adverse effects of reversible hearing loss (HL) on speech and language development, even though the risk of complications such as tube extrusion and iatrogenic cholesteatoma needs to be taken into account [7]. Some previous reports have emphasized the importance and safety of early placement of a VT to optimize hearing outcome in CLP patients [8]. Moreover, adequate hearing function plays a significant role in articulation training after palatoplasty by helping the patient to hear others’ voices and pronounce words correctly by hearing their own voice during a critical period of growth and development [9,10]. It has also been suggested that treatment with early VT insertion provides a hearing benefit in children younger than 4 years of age, but no significant difference after 4 years [11]. Nevertheless, there is no consensus regarding the indications for and the timing of VT insertion [12]. Some authors did not find significant differences in CLP patients treated with VT, and suggested that middle ear status improved as patients grew older [13]. Others reported that age at palatoplasty was the main prognostic factor influencing hearing outcome in CLP children [14].

In this retrospective study, we evaluated a series of consecutive CLP patients operated on at a regional referral center. The aim was to search for clinical and surgical (i.e., VT insertion) characteristics that influence hearing status.

## 2. Materials and Methods

### 2.1. Patients and Procedures

The study was conducted in accordance with the principles of the Helsinki Declaration and was approved by the internal ethics committee. Data were examined in agreement with the Italian privacy and sensible data laws. In this retrospective study, we analyzed the records of consecutive CLP patients referred to Vicenza Civil Hospital (Italy), which is a member of the European Reference Network CRANIO.

Patients were included in the study based on the following criteria: (1) congenital unilateral or bilateral CLP; (2) treatment with the protocol of the Regional Hospital of Vicenza; (3) available audiometric data during follow-up. Patients were excluded if they had syndromic CLP, isolated cleft lip, or isolate cleft palate.

All patients had a surgical protocol in accordance with the Regional Hospital of Vicenza. The chronological order of the treatment, according to the following protocol: (i) at birth: placement of a passive palate plate; (ii) at 4 months of age: soft palate surgery; (iii) at 6 months of age: unilateral or bilateral lip and nose repair; (iv) at 18 to 20 months of age: hard palate surgery with/without VT insertion; (v) at 9 to 12 years: alveolar bone graft; (vi) at 5 years and 15 years: orthodontic treatment and follow-up visits; and (vii) at 18 years of age: orthognathic surgery when needed, rhinoplasty, soft tissue deformity correction.

Patients were referred to first ENT evaluation prior to hard palate surgery (18–20 months). ENT surgeons decided on VT insertion during palate surgery (i.e., early VT), or for follow-up, according to patients’ clinical examination. ENT follow-up visits were scheduled subsequently according to the patient’s needs, at least once every year. Hearing assessment was performed prior to hard palate surgery and during follow-up. We used behavioral procedures including visual reinforcement audiometry (VRA) for children between approximately 6–18 months of age, and conditioned play audiometry (CPA) for older children. When patient collaboration increased, we performed pure-tone audiometry in a silent cabin including bone-conduction (BC) thresholds at 0.5, 1, 2, and 4 kHz, and air-conduction (AC) thresholds at 0.25, 0.5, 1, 2, 4, and 8 kHz, for both ears [15]. When OME was found during follow-up, patients underwent VT insertion (i.e., late VT).

For the outcome variable, we considered the presence of hearing loss at the last available follow-up visit. Hearing loss was defined as an air conduction pure-tone average (PTA) >20 dB (calculated as a 4-frequency average at 0.5, 1, 2, and 4 kHz) [16]. The degree of hearing impairment was defined by the PTA and was classified as mild (PTA 21–40 dB HL), moderate (PTA 41–70 dB HL), severe (PTA 71–95 dB HL), and profound (PTA > 95 dB HL), as previously reported [16].

### 2.2. Statistical Analysis

We used the Fisher exact test, the Mann–Whitney U test, and the chi-square test as appropriate. For every significant association disclosed by the Fisher exact test, we calculated an odds ratio (OR). When necessary, continuous variables were dichotomized according to the median value, as previously reported [17]. A multivariate logistic model was constructed, adding only the clinical parameters with a *p* value ≤ 0.1, as disclosed by Fisher exact test in univariate analysis, according to previous multivariate modeling experience [18]. The results were expressed as ORs, *p* values, and 95% confidence intervals (CIs). During the analysis, the model was checked for multicollinearity with a variance inflation factor test. A *p* value < 0.05 was considered significant. The quality of the model was assessed with Pearson chi-squared test, a non-significant result (*p* ≥ 0.05) indicating a good fit of the model to the data. The Social Sciences version 17 statistical package (SPSS Inc., Chicago, IL, USA) was used for all analyses.

## 3. Results

In the present retrospective study, we included 285 consecutive CLP patients operated on at the Regional Hospital of Vicenza. We found 109 female and 176 male patients; the mean age at last follow-up was 16.2 years. Unilateral CLP was found in 249 cases, while bilateral CLP in 36. Early VTs were applied in 75 (26.3%) patients (bilateral in 69, and unilateral in 6 cases) at time of palate surgery. Late VTs were applied in 69 (24.2%) children (bilateral in 51 cases, unilateral in 18) at a mean age of 6.7 years old. Hearing loss (PTA > 20 dB) was found in 114 (40%) CLP patients at last available follow-up (mild hearing loss in 96 patients, moderate in 18).

In Table 1, we report the demographic and clinical characteristics of patients according to hearing status. Persistent tympanic membrane perforation was found in 6 (8%) cases after early VT and in 5 (7%) after late VT. We found three cases of cholesteatoma in our children, due to tympanic membrane retraction.

Using the Fisher exact test, we performed a univariate analysis with dichotomized variables (Table 2), in order to discover an association with the outcome (i.e., presence of hearing loss at last follow-up). We found a significant association for the absence of early VT insertion (*p* = 0.0001; OR 19.25) and late VT insertion (*p* = 0.0001; OR 23.1). Univariate analysis disclosed three characteristics with a *p* value of ≤ 0.1 in the Fisher exact test: unilateral CLP, the absence of early VT, and late VT. Using these three variables in the model, the variance inflation factor test detected multicollinearity in the model. In fact, when early VTs were not inserted, there was a high risk of late VT (Fisher exact test, *p* = 0.002; OR 13.6). The model with two variables (unilateral CLP and the absence of early VT) showed no multicollinearity. Although unilateral CLP (OR = 1.63; *p* = 0.25, CI95% 0.69–4.17) was not associated with the outcome, the absence of early VT insertion (OR = 18.2; *p* = 0.0003, CI95% 1.46–35.90) was an independent prognostic factor of hearing loss in CLP patients. The results of the Pearson chi-squared test (*p* = 0.55) showed a good fitting of the empirical model to real data.

## 4. Discussion

Hearing loss has been thought to have a significant impact on children’s language and psychosocial development [19]. Therefore, the rapid and adequate treatment of hearing loss should be mandatory [3]. The overall rate of HL in our group was 40%, analogous with other retrospective studies [2]. Although early hearing rehabilitative intervention has gained some support in patients with CLP, a more conservative approach is favored by other groups because of the risk of morbidity associated with repeated TT insertion, including persistent perforations, myringosclerosis, and cholesteatoma [20]. Persistent perforation after VT was low in our case series (7.5%). Regarding the risk of cholesteatoma after VT in cleft palate patients, a study of 116 patients with a 72-month follow-up indicated that VT had no influence on the development of cholesteatoma [21]. Similarly, we found only three cases of cholesteatoma due to tympanic membrane retraction. Although VT placement can be beneficial in the treatment of OME to prevent short-term hearing loss, there is no consensus regarding the indication criteria or timing of VT insertion [12].

In this retrospective study, we evaluated a series of 285 consecutive CLP patients operated on at a regional referral center. The aim was to search for clinical and surgical (i.e., VT insertion) characteristics that influenced hearing status. At univariate and multivariate analysis, we found that only the absence of early VT insertion (OR = 18.2; *p* = 0.0003) was an independent prognostic factor of hearing loss in CLP patients. Furthermore, when early VTs were not inserted, there was a high risk of late VT (*p* = 0.002; OR 13.6). Iemura-Kashiwagi and colleagues [3] evaluated 75 CP patients. They concluded that VT insertion at the same time as palatoplasty may reduce the number of times the patient requires general anesthesia and maintain good middle ear condition during the period of language acquisition between 1 and 3 years of age [3]. According to a retrospective study on 97 CLP patients conducted by Klockars et al. [8], early tympanostomy tube placement should always be considered; VT combined with early soft palate closure (at four months of age) reduced the frequency of OME [8]. In their systematic review, Kuo et al. [22] investigated the effectiveness of VT for OME in children with CP. Compared with conservative forms of management (e.g., watchful waiting), VT insertion has been shown to be beneficial to the recovery of hearing in children with CP and OME [22]. Nevertheless, the authors concluded that results were based on underpowered cohort studies and very low-strength evidence [22]. Another systematic review aimed to examine the effectiveness and potential complications of VT placement prior to palatoplasty in infants with CLP [23]. The authors found that early VT in CLP led to similar speech and audiology outcomes to non-CLP children undergoing ventilation tube insertion, and better outcomes than those children with CLP having later VT insertion [23]. According to Stanton et al. [24], many studies found no difference in hearing or speech between early and late VT. The study by Tengroth et al. [4] found no correlation between VT insertions and hearing outcome of CLP children [4]. However, the authors noted that there may be confounding factors that could have affected their results, such as differences in severity of OME, or other medical conditions that may impact hearing or speech development [4]. According to recent systematic review, additional studies are needed to provide stronger evidence regarding VT timing in CLP [24].

Most studies on VT in CLP patients have significant limitations, including small sample size or not reporting on primary outcome [24]. Imbery et al. [25] studied more than 300 CLP patients, but they did not investigate the outcome in relation to early VT insertion. To the best of our knowledge, the present study is one of the largest to investigate this matter. We found a high risk of hearing loss in CLP patients who did not receive early VT. The main limitation of our investigation is the retrospective design.

## 5. Conclusions

According to our results, the absence of VT insertion at the time of hard palate surgery was an independent prognostic risk factor of hearing loss in CLP patients. Early VT placement in CLP patients may prevent hearing loss and related consequences. These findings should be tested in large, randomized clinical trials.

## Figures and Tables

**Table 1:** Demographic and clinical characteristics of cleft lip and palate (CLP) patients according to hearing status at last available follow-up.

	Normal Hearing	Hearing Loss
Sex (female/male)	58/96	51/80
Age in years (SD)	17.2 (5.8)	15.7 (6.7)
Unilateral CLP (patients)	144	105
Bilateral CLP (patients)	27	9
Absence of early VT (patients)	108	102
Early VT (patients)	63	12
Late VT (patients)	18	51
Total Patients	171	114

Abbreviations: CLP (cleft lip and palate); SD (standard deviation), VT (ventilation tube).

**Table 2:** Univariate and multivariate analyses of the outcome (presence of hearing loss at last follow-up) of cleft lip and palate patients.

	Univariate Analysis	Multivariate Analysis *
Female gender	0.92	NA
Age > 8.4 years	0.42	NA
Unilateral CLP	0.07	0.06
Absence of early VT	0.0001	0.0001
Late VT	0.0001	Excluded **

* Only the clinical parameters with a *p* value ≤ 0.1 disclosed by Fisher exact test in univariate analysis were included; ** Excluded because the variance inflation factor test detected multicollinearity. Abbreviations: CLP (cleft lip and palate); NA (not applicable); VT (ventilation tube).

## Data Availability

Data are available upon request to the corresponding author.
